# Longitudinal assessment of right ventricular structure and function by cardiovascular magnetic resonance in breast cancer patients treated with trastuzumab: a prospective observational study

**DOI:** 10.1186/s12968-017-0356-4

**Published:** 2017-04-10

**Authors:** Ashita Barthur, Christine Brezden-Masley, Kim A. Connelly, Vinita Dhir, Kelvin K. W. Chan, Rashida Haq, Anish Kirpalani, Joseph J. Barfett, Laura Jimenez-Juan, Gauri R. Karur, Djeven P. Deva, Andrew T. Yan

**Affiliations:** 1 Department of Medical Imaging, St. Michael’s Hospital, University of Toronto, Toronto, ON Canada; 2 Division of Hematology/Oncology, St. Michael’s Hospital, University of Toronto, Toronto, ON Canada; 3 Keenan Research Centre, Li Ka Shing Knowledge Institute, St. Michael’s Hospital, University of Toronto, Toronto, ON Canada; 4 Terrence Donnelly Heart Centre, St. Michael’s Hospital, University of Toronto, Toronto, ON Canada; 5 Division of Cardiology, St. Michael’s Hospital, University of Toronto, Toronto, ON Canada; 6 Sunnybrook Health Sciences Centre, University of Toronto, Cancer Care Ontario, Canadian Center for Applied Research in Cancer Control, Toronto, ON Canada; 7 Department of Medical Imaging, Sunnybrook Health Sciences Centre, University of Toronto, Toronto, ON Canada

**Keywords:** Right ventricle, Cardiovascular magnetic resonance, Cardiotoxicity, Trastuzumab

## Abstract

**Background:**

There are limited data on the effects of trastuzumab on the right ventricle (RV). Therefore, we sought to evaluate the temporal changes in right ventricular (RV) structure and function as measured by cardiovascular magnetic resonance (CMR), and their relationship with left ventricular (LV) structure and function in breast cancer patients treated with trastuzumab.

**Methods:**

Prospective, longitudinal, observational study involving 41 women with HER2+ breast cancer who underwent serial CMR at baseline, 6, 12, and 18 months after initiation of trastuzumab. A single blinded observer measured RV parameters on de-identified CMRs in a random order. Linear mixed models were used to investigate temporal changes in RV parameters.

**Results:**

Of the 41 women (age 52 ± 11 years), only one patient experienced trastuzumab-induced cardiotoxicity. Compared to baseline, there were small but significant increases in the RV end-diastolic volume at 6 months (*p* = 0.002) and RV end-systolic volume at 6 and 12 months (*p* < 0.001 for both), but not at 18 months (*p* = 0.82 and 0.13 respectively). RV ejection fraction (RVEF), when compared to baseline (58.3%, 95% CI 57.1–59.5%), showed corresponding decreases at 6 months (53.9%, 95% CI 52.5–55.4%, *p* < 0.001) and 12 months (55%, 95% CI 53.8–56.2%, *p* < 0.001) that recovered at 18 months (56.6%, 95% CI 55.1–58.0%, *p* = 0.08). Although the temporal pattern of changes in LVEF and RVEF were similar, there was no significant correlation between RVEF and LVEF at baseline (*r* = 0.29, *p* = 0.07) or between their changes at 6 months (*r* = 0.24, *p* = 0.17).

**Conclusion:**

In patients receiving trastuzumab without overt cardiotoxicity, there is a subtle but significant deleterious effect on RV structure and function that recover at 18 months, which can be detected by CMR. Furthermore, monitoring of LVEF alone may not be sufficient in detecting early RV injury. These novel findings provide further support for CMR in monitoring early cardiotoxicity.

**Trial registration:**

ClinicalTrials.gov Identifier: NCT01022086. Date of registration: November 27, 2009.

## Background

Breast cancer is the most common cancer and second most common cause of cancer death among women in North America [1, 2]. At least 20% of invasive breast cancers demonstrate human epidermal growth factor receptor type 2 (HER2) over-expression which adversely affects prognosis [3]. Recent guidelines recommend evaluation of HER2 status in all invasive breast cancers at diagnosis or recurrence for consideration of trastuzumab therapy [4]. Cardiac dysfunction, defined as a symptomatic decline in left ventricular ejection function (LVEF) of >5% from baseline or an asymptomatic reduction of >10% from baseline to <55%, has been recognized as a potential dose-limiting toxicity of trastuzumab therapy [5]. Cardiac dysfunction occurs in up to 34% patients on trastuzumab and anthracyclines leading to adverse cardiac events [6], and is mediated by HER2 receptor inhibition in cardiomyocytes [7, 8]. RV dysfunction is known to be a poor prognosticator in a variety of other cardiovascular diseases [9–16]. However, there are limited data regarding trastuzumab induced toxicity affecting the right ventricle (RV) [17, 18]. The initial studies on cardiotoxic effects in breast cancer patients utilized radionuclide ventriculography [19, 20] and echocardiography [21–24], which have limited accuracy in evaluating RV owing to its shape and position, in addition to inherent drawbacks of each modality [25].

## Methods

### Aim

To evaluate the temporal changes in RV structure and function as measured by cardiovascular magnetic resonance (CMR) over 18 months, and to examine the relationship between the LV and RV structure and function in breast cancer patients treated with trastuzumab.

### Study protocol

This prospective cohort study comprised of 41 consecutive eligible women from the medical oncology departments of 2 tertiary care hospitals, who were newly diagnosed with HER2 positive breast cancer between Jan 2010 and Dec 2013. The study was approved by the Research and Ethics Boards of both hospitals. Written informed consent was obtained from each participant. We included women older than 18 years; with histologically confirmed invasive breast cancer and HER 2 over-expression; planned to be treated with trastuzumab; with a baseline LVEF ≥50% by radionuclide ventriculography. Those with previous treatment with trastuzumab, other anti-HER2 agent, pre-existing symptomatic heart failure (NYHA Class III or IV), recent acute coronary syndrome or coronary revascularization within the last 6 months, permanent atrial fibrillation, contraindications to MRI and pregnant and/or nursing patients were excluded.

All participants were scheduled for CMR at baseline (prior to the initiation of trastuzumab therapy) and at 6, 12 and 18 months following initiation of therapy. Blood samples were obtained for measurements of serum NT-BNP and high-sensitivity troponin I (hs-TnI) as biomarkers of cardiotoxicity.

### Trastuzumab and chemotherapy regimen

Chemotherapy consisted of anthracycline or non-anthracycline regimen at the discretion of the treating oncologist. Participants received 18 cycles (12 months) of trastuzumab therapy.

### Cardiotoxicity

All patients underwent serial radionuclide ventriculography for monitoring of LVEF, as part of standard of care. In the event of cardiotoxicity based on LVEF measurement by radionuclide ventriculography, trastuzumab treatment was withheld for 3 weeks [26]. After 3 weeks, if the repeat radionuclide ventriculography scan showed improvement in LVEF, then trastuzumab was re-initiated. Both the medical oncologist and patient were blinded to the results of the CMR.

### CMR

CMR was performed at baseline, 6, 12 and 18 months. All CMR was performed with a 1.5 T scanner (Intera, Philips Medical Systems, Best, the Netherlands, or GE Signa Excite Cv, Milwaukee, WI) using a phased-array cardiac coil and retrospective electrocardiographic gating. Standard protocols using validated, commercially available sequences were used. Images were obtained with breath-hold at end-expiration. Segmented, balanced steady-state free-precession sequence was used for cine acquisition with the following typical parameters: TR 4 ms, TE 2 ms, slice thickness 8 mm, field of view 320–330 x 320–330 mm, matrix size 256x196, temporal resolution of <40 ms (depending on the heart rate) and flip angle 50 degrees. Following administration of intravenous gadolinium contrast, phase sensitive inversion recovery sequences were obtained. CMR scans were analyzed with CVi 42 software (Circle Cardiovascular, Calgary, Alberta, Canada).

A single blinded experienced reader measured all RV parameters on de-identified CMR scans in a random order. LV measurements and late gadolinium enhancement (LGE) assessment were made independently by another blinded experienced reader.

Both the LV and RV were analyzed only by volumetric measurements on the short-axis cine steady state free precision images. The LV and RV were contoured according to the current practice at our institution using semi-automated tracing of the endocardial borders at end diastole and end systole, which corresponded to the cardiac phases with the largest and smallest ventricular volumes, respectively. Papillary muscles and trabeculations were considered part of the blood pool and not as a part of the RV mass. RV end-diastolic and end-systolic volumes (RVEDV and RVESV) were determined by summing the volume across slices without geometric assumptions, and EF is calculated as (EDV-ESV)/EDV x100% [27]. The normal reference range for RVEF in women was defined as 51–71% [28].

### Statistical analysis

The sample size calculation was based on the detection of clinically meaningful changes in LV volume and LVEF, between baseline and 12 months, using the simple paired *t*-test approach. 38 subjects would provide at least 95% power to detect a 10-ml mean change in LV volumes, or a 3% mean change in LVEF. This sample size of 38 patients also achieves 95% power to detect an intraclass correlation of 0.80 between LVEF measurements by CMR and radionuclide ventriculography (when the null hypothesis is 0.50) at a significance level of 0.05. However, because the impact of trastuzumab on RV volume and RVEF changes were not known and our study is the first study to longitudinally assess the changes in RV parameters in breast cancer patients treated with trastuzumab over 18 months, the sample size was not based on expected changes in RV volume or RVEF. Mean and standard deviation (or median and interquartile range) are reported for continuous variables. Mixed linear models were performed to test for longitudinal changes in RV and LV parameters over time. This method has the advantage of allowing all available data over time to be analyzed, unlike repeated measures ANOVA which allows only complete case analysis. We selected the unstructured covariance based on the Akaike’s information criterion. Sidak adjustment was utilized for pairwise comparisons of the three time points (6, 12, 18 months) with the baseline measurement. We also tested for potential interaction between anthracycline use and time in the mixed linear model. We calculated the Pearson correlation coefficients between RV and LV parameters. To examine the non-linear relationship between biomarker and RVEF, we used the non-parametric Kendall tau-b (τ) correlation test. To explore whether cardiovascular risk factors might impact on RV volume and RVEF, we compared the changes in RVEDV, RVESV and RVEF at 6 months in the group with versus the group without any cardiovascular risk factors (hypertension, diabetes, dyslipidemia or smoking). To assess intra-observer reproducibility, we calculated the intra-class correlation coefficients for RVEDV and RVESV. A two-sided *p*-value <0.05 was defined as significant. Statistical analysis was performed using SPSS version 22 (IBM Corp., Armonk, NY).

## Results

In total, 41 women who received trastuzumab underwent CMR at baseline; 33 of them completed the 18 months follow up, and the remaining 8 patients did not undergo all the CMRs either due to tissue expanders, change in treatment or personal reasons. Table 1 shows the baseline characteristics of the study population. Amongst these, 23 patients received anthracycline based chemotherapy. There were no patients with heart failure at baseline.

**Table 1:** Baseline characteristics of the study population

	*n* (%), Total *n* = 41
Demographics	
Age	52 ± 11
BMI, kg/m2	26.8 ± 6.3
Cardiovascular risk factors	
Hypertension	10 (24.4%)
Diabetes	4 (9.8%)
Hypercholesterolemia	3 (7.3%)
Coronary artery disease	1 (2.4%)
Smoking	10 (24.4%)
Medications:	
Beta-blocker	3 (9%)
ACE inhibitor	3 (9%)
NYHA class III/IV	0
Cancer-related variables	
Breast cancer site	
Left	27 (65.9%)
Stage of disease	
Early	27 (68%)
Locally invasive	13 (32%)
Type of surgery	
Breast-conserving surgery	22 (53.7%)
Mastectomy	19 (46.3%)
Chemotherapeutic regimen	
Anthracycline based	23 (56.1%)
Left sided radiation	12 (29.3)
Biomarkers:	
High sensitivity-troponin I, ng/mL (*n* = 37)^a^	<0.006 (<0.006–0.012)
NT-BNP, ng/mL (*n* = 39)^a^	57 (33–128)
LV parameters	
LVEDV, ml	129.7 ± 24.9
LVESV, ml	51.5 ± 12
LVEF, %	60.4 ± 4.2
RV parameters	
RVEDV, ml	121 ± 24
RVESV, ml	51 ± 11
RVEF, %	58.3 ± 3.8

Data presented as frequency (percentages) or mean ± standard deviation unless otherwise specified

^a^median (interquartile range)

Only 1 patient experienced cardiotoxicity for which trastuzumab was subsequently withheld for one cycle. No patient developed new clinical heart failure or died due to heart failure during the study follow up.

**Fig. 1:**
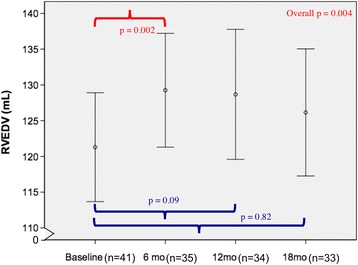
Longitudinal measurements of RVEDV over 18 months. There were significant changes in RVEDV over time (*p* = 0.004). By Sidak-adjusted pairwise comparisons with the baseline, the mean RVEDV showed a small but significant increase at 6 months (*p* = 0.002), that was no longer significant at 18 months (*p* = 0.82). Data shown as mean and 95% confidence intervals (*vertical bars*)

**Fig. 2:**
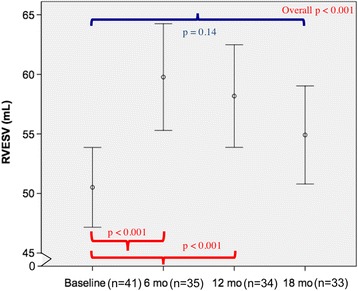
Longitudinal measurements of RVESV over 18 months. There were significant changes in RVESV over time (*p* < 0.001). By Sidak-adjusted pairwise comparisons with the baseline, the mean RVESV showed a small but significant increase at 6 and 12 months (both *p* < 0.001), that was no longer significant at 18 months (*p* = 0.14). Data shown as mean and 95% confidence intervals (*vertical bars*)

**Fig. 3:**
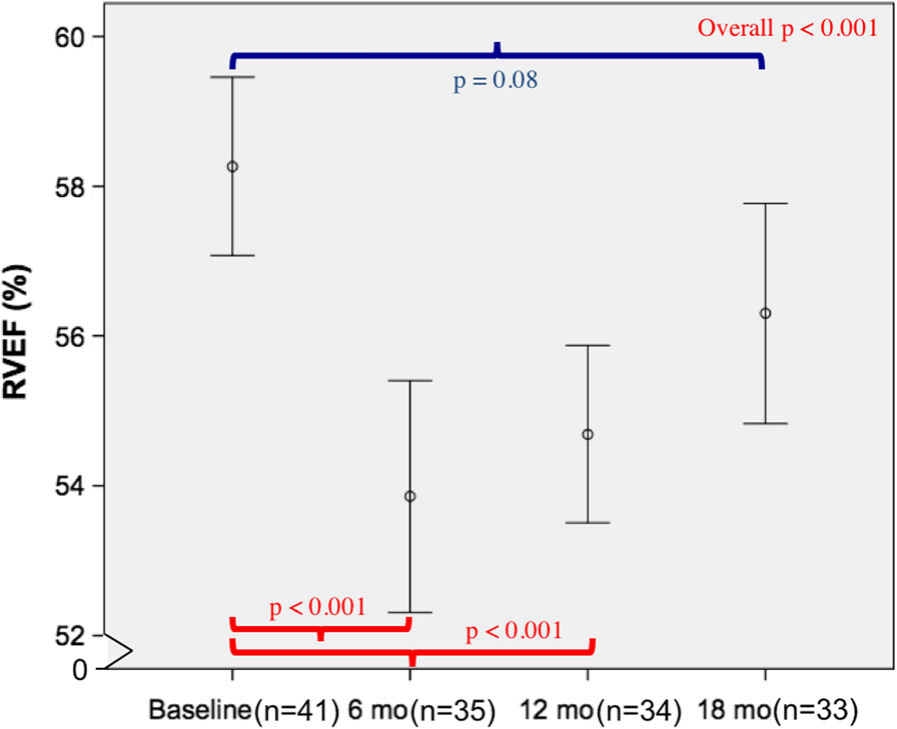
Longitudinal measurements of RVEF over 18 months. There were significant changes in RVEF over time (*p* < 0.001). By Sidak-adjusted pairwise comparisons with the baseline, the mean RVEF showed a small but significant decrease at 6 and 12 months (*p* < 0.001 for both), that was no longer significant at 18 months (*p* = 0.08). Data shown as mean and 95% confidence intervals (*vertical bars*)

RVEDV, RVESV, and RVEF changed significantly over the course of the study (Figs. 1, 2 and 3). Although the mean RVEF remained normal (≥51%) throughout the study, 8/35 patients (23%) showed a decline in RVEF to less than the lower limits of normal (<51%) at 6 months; only 2 of these patients had a decrease from baseline of more than 10%. At 12 months, 4/34 (12%) patients had an RVEF below the lower limit of normal, with only one patient demonstrating a persistent drop in RVEF of more than 10% when compared to baseline.

By pairwise comparisons with the baseline, there was a significant increase in RVEDV only at 6 months (129.9 ml, 95% CI 122–137.7 ml, *p* = 0.002) but not at 12 months (127 ml, 95% CI 118.3–135.7 ml, *p* = 0.09) and 18 months (123.3 ml, 95% CI 115–131.7 ml, *p* = 0.82) (Fig. 1), whereas the increase in RVESV were significant at both 6 months (59.9 ml, 95% CI 55.3–64.4 ml, *p* < 0.001) and 12 months (56.7 ml, 95% CI 52.6–60.8 ml, *p* < 0.001) but not at 18 months (53.4 ml, 95% CI 49.5–57.3 ml, *p* = 0.14) (Fig. 2). Analogous to the changes in RVESV, we noted a significant decline in the mean RVEF at 6 months (53.9%, 95% CI 52.5–55.4%, *p* < 0.001) and 12 months (55%, 95% CI 53.8–56.2%, *p* < 0.001), which became non-significant at 18 months (56.6%, 95% CI 55.1–58.0%, *p* = 0.08) (Fig. 3). When indexed RV volumes were analysed, the overall temporal changes and pattern remained the same (data not shown).

**Fig. 4:**
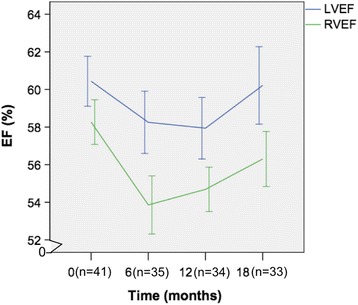
Relationship between LVEF and RVEF over 18 months. The changes in LVEF and RVEF paralleled each other over time. Both the LVEF and RVEF showed significant changes at 6 and 12 months that recover at 18 months

**Fig. 5:**
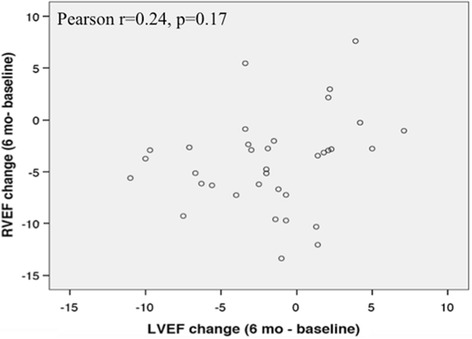
Relationship between changes in RVEF and LVEF over 6 months

The mean LVEF at baseline was normal (60.4 ± 4.2%). Although the mean LVEF remained normal throughout the study, there was a significant decrease in LVEF at 6 and 12 months (58.3 ± 5.1%, 57.9 ± 4.8%, respectively, both *p* < 0.05) compared to baseline. The changes in LVEF and RVEF paralleled each other during treatment; both were significantly lower at 6 and 12 months and both recovered by 18 months (Fig. 4). Overall, RVEF and LVEF showed weak and non-significant correlation at baseline (*r* = 0.29, *p* = 0.07). Likewise, the changes in LVEF and RVEF over 6 months showed only a weak and non-significant correlation (*r* = 0.24, *p* = 0.17, Fig. 5).

There was no significant interaction between anthracycline and time for RVEF (*p* = 0.12), RVEDV (*p* = 0.52), and RVESV (*p* = 0.25). This suggests that the temporal changes in RVEF, RVEDV, and RVESV were similar between the anthracycline and non-anthracycline groups.

The median (interquartile range) for NT-BNP was 57(33–128) at baseline, 39(30.3–86.5) at 6 months, 47.5(29.3–74.8) at 12 months and 61(36.8–111.8) at 18 months. There was no significant correlation between changes in NT-BNP and RVEF at 6 months (τ = 0.11, *p* = 0.42). There was no significant change in the hs-TnI [median(interquartile range)] from baseline <0.006 ng/ml (<0.006–0.012) to 6 months <0.006 ng/ml(<0.006– < 0.006) or 12 months <0.006 ng/ml(<0.006– < 0.006).

There was no significant difference in changes in RVEF, RVEDV or RVESV at 6 months between the group with any cardiovascular risk factor (*n* = 15) and with no risk factors (*n* = 20).

Only 2 patients demonstrated LGE. One patient had an ischemic pattern of LGE (attributable to a previous myocardial infarction) which was seen at baseline and did not change or develop new focus of LGE during the study. The other patient showed a non-specific inferior RV hinge point LGE at 12 months that was not subsequently evident at 18 months.

There was excellent intra-observer reproducibility; the intra-class correlation coefficients for absolute agreement were 0.97 for RVEDV and 0.98 for RVESV (both *p* < 0.001).

## Discussion

In this prospective longitudinal study of 41 consecutive patients treated with trastuzumab over 18 months with rigorous blinding strategies, we found small, but significant increases in RV volumes, with a corresponding subtle reduction in RVEF during trastuzumab therapy. These changes were no longer significant after completion of trastuzumab therapy (18 months). The pattern of temporal changes in LVEF and RVEF paralleled each other but showed only weak and non-significant correlation. By demonstrating that CMR can detect even subclinical RV changes in breast cancer patients treated with trastuzumab, our study provides further support for the use of CMR in research and clinical practice.

The cardiotoxic effect of trastuzumab in breast cancer patients has been explained on the basis of HER2 receptors in cardiomyocytes. Trastuzumab blocks these HER2 receptors in the cardiomyocytes rendering both RV and LV vulnerable to type II or reversible cardiotoxicity [7, 8]. Theoretically, the thinner structure of the RV with lesser myofibrils may make it more vulnerable to cardiotoxicity induced by trastuzumab [18, 24]. A decrease in RV function and RV hypertrophy have been associated with an increased risk of morbidity and mortality in other diseases such as heart failure, post myocardial infarction, congenital heart disease and dilated cardiomyopathy [12–16]. Furthermore, changes in RV function over time have been shown to confer incremental prognostic value in dilated cardiomyopathy [16].

RV dysfunction has been demonstrated as RV wall motion abnormalities [20] or functional abnormalities [18, 24] in patients receiving anthracyclines and trastuzumab. Initial studies evaluating trastuzumab induced RV dysfunction utilized 2D-echocardiography, which has limited accuracy in assessing RV size and function owing to its shape and position, and other inherent drawbacks including operator dependency and need for a good acoustic window for accurate measurements [29]. Furthermore, the results based on these echocardiography studies have been inconsistent [30]. Lange et al. used 2D-echocardiography at baseline, 3 and 6 months after trastuzumab which showed no difference in global RV function [21], while Kilicaslan et al. demonstrated a reduction in RV function at 6 months, similar to our results [22]. Another 2D-echocardiography based study by Calleja et al. retrospectively analyzed 30 breast cancer patients with cardiotoxicity who received trastuzumab [23]. RV dysfunction based on RV fractional area change, RV global longitudinal strain and RV free wall peak systolic longitudinal strain was seen in 40% of their patients, whereas it was seen in 23% of our study participants. Most of these previous studies utilized conventional, M mode, and Doppler echocardiography derived measurements such as tricuspid annular plane systolic excursion, RV fractional area change, RV myocardial performance index to evaluate RV function. 2D-echocardiography based RV strain, although angle independent and gives information regarding global and regional strain, provides only longitudinal strain information on the apical 4 chamber view. Another important inherent limitation is loss of speckles due to motion outside the imaging plane and excessive motion of the RV lateral wall. Also, the use of different techniques, intervendor variability and lack of reference values make CMR derived RV parameters likely more robust [31]. Although 3D-echocardiography based RV strain has been used to assess RV regional wall motion abnormality in patients with tetralogy of Fallot, there was no validation of this technique with references such as 2D-echocardiography based strain or CMR [32]. Other studies have shown newer techniques like 3D-echocardiography based strain parameters to have relatively low reproducibility in comparison with 2D-echocardiography and CMR [31]. CMR has the advantage of excellent endocardial definition, accurate volumetric measurements with no geometric assumptions, and is therefore the reference standard for RV volumes and RVEF.

To the best of our knowledge, there have been only two published studies utilizing CMR to evaluate the effects of trastuzumab therapy on the RV [17, 18]. However, it is difficult to draw conclusions from these previous studies as trastuzumab was only utilized in a third (15/46) of patients in one study [18], while only 9 of 16 patients in the other study were evaluated by CMR [17]. Furthermore, neither study had follow-up beyond 1 year. The RVEDV, RVESV and RVEF remained unchanged over the time period in the small study of 9 patients [17]. In contrast, Grover et al. demonstrated a significant decline in RV function at 4 months that persisted at 12 months; thereafter RV function was not re-assessed [18]. Our results supported their findings of decline in RV function during treatment. Importantly, we have also demonstrated a recovery of RVEF at 18 months (6 months post completion of trastuzumab therapy) which has not been reported previously. Furthermore, to the best of our knowledge, no study has reported the relationship between the temporal changes in the RVEF and LVEF among patients receiving trastuzumab. Our study revealed only a weak and non-significant correlation between change in LVEF and RVEF over time. This suggests that early RV changes cannot be reliably detected by monitoring LV *alone*.

Previous studies have assessed longitudinal changes in NT-BNP but not relationship between NT-BNP and RVEF. Similar to results by Grover et al. and Nakano et al., we did not find a significant change in NT-BNP before and after initiation of trastuzumab therapy. In addition, we observed no significant correlation between NT-BNP and RVEF and RV volumes. This novel finding implies that NT-BNP may not be a useful biomarker for subclinical changes in RV function. This may be explained by the only subtle changes in RV structure and function in our participants, who were not selected based on presence of overt cardiotoxicity. It is also plausible that most of the BNP originates from the LV rather than the RV. These findings further support the use of CMR as a unique non-invasive imaging modality to detect the early or subclinical RV changes, for which biomarkers may not be a reliable surrogate.

Several study limitations should be noted. Our study cohort was relatively small, but it is the largest longitudinal study published to date of trastuzumab-treated patients using CMR, and adds to the existing literature which has been scarce. A few patients did not complete all 4 scheduled CMR scans over 18 months, but the linear mixed model allowed all available CMR data to be analyzed. We did not evaluate short term (1–4 months) and long term effects (>18 months) on the RV. Nevertheless, the aim of our study was to accurately assess the temporal changes in RV over 18 months, and compare it to the LV, which is unique to our study. We utilized RVEF and not RV strain to evaluate RV function due to its thin free wall. Our study did not have adequate power to examine the significance of cardiovascular risk factors and to assess clinical outcomes, but RVEF is expected to carry prognostic value as in other disease states [12–16]. While we speculate that the small subclinical RV changes that we observed in our cohort *without* overt cardiotoxicity are likely not clinically significant, future studies are needed to precisely determine the prognostic and therapeutic implications of the *spectrum and progression* of RV changes. Importantly, this study demonstrates the potential of CMR to detect the *early* and subtle changes in RV function, even *before* the development of overt cardiotoxicity.

As a multicenter study with utilization of two vendors for the performance of CMR and broader representation of patients, our study has greater generalizability in routine clinical practice in the real world. Furthermore, in contrast to previous studies, this prospective cohort study employed a rigorous blinding strategy, in which a single observer was blinded to all the clinical data, time of occurrence, and the LV parameters, thereby reducing bias. The demonstration of the detrimental effect of trastuzumab on the RV during the course of treatment, with recovery 6 months post completion of treatment, is novel and biologically plausible. The observation of weak and non-significant correlation between the pattern of temporal changes in LVEF and RVEF has also not been reported in the existing literature. Importantly, it illustrates that monitoring of LVEF alone may not reveal subtle changes in RV structure and function during trastuzumab therapy. Our findings support the recent American Society of Echocardiography guidelines [33] which recommend routine follow up of RV and LV during cancer therapy. An accurate and reproducible imaging technique such as CMR may be particularly valuable in this setting.

Our study raises several important questions for future research. Although it remains to be determined whether RV dysfunction provides incremental prognostic information specifically in this patient population, the current definition of cardiotoxicity (arbitrarily based on a decline in LVEF only) may not always adequately reflect cardiac injury. While isolated RVEF decline may not warrant treatment discontinuation, its prevalence and clinical significance need to be elucidated. Currently, AHA/ACC practice guidelines for heart failure recommend angiotensin converting enzyme inhibitors or angiotensin receptor blockers and beta-blockers for asymptomatic LV dysfunction (stage B heart failure). It would be important to determine whether these neurohormonal modulators would also be cardioprotective for the RV. With emerging sequences such as T1 and T2 mapping and extracellular volume assessment, there is potential for CMR to provide comprehensive assessment of cardiotoxicity. Further studies should determine whether these “imaging biomarkers” may furnish greater or incremental utility beyond the serum biomarkers (TnI and NT-BNP) in monitoring cardiotoxicity.

## Conclusion

There were small but significant increases in RV volumes, with a corresponding subtle reduction in RVEF during trastuzumab therapy. The pattern of temporal changes in LVEF and RVEF paralleled each other but revealed only weak and non-significant correlation. In addition to accurate assessment of LV function and scar, CMR is a unique, safe, and promising modality to reliably monitor deterioration in RV function. Further studies are required to determine its prognostic and therapeutic implications.

## Abbreviations

CI: Confidence interval
CMR: Cardiovascular magnetic resonance
EDV: End-diastolic volume
EF: Ejection fraction
ESV: End-systolic volume
HER2: Human epidermal growth factor receptor 2
hs-TnI: High-sensitivity troponin I
LGE: Late gadolinium enhancement
LV: Left ventricle
NT-BNP: N-terminal prohormone of brain natriuretic peptide
NYHA: New York Heart Association (NYHA)
RV: Right ventricle
